# Associations of Patients with Pericardial Effusion Secondary to Light-Chain or Transthyretin Amyloidosis- A Systematic Review

**DOI:** 10.2174/011573403X280737240221060630

**Published:** 2024-03-08

**Authors:** Nismat Javed, Kirit Singh, Justin Shirah, Timothy J. Vittorio

**Affiliations:** 1 Department of Internal Medicine, BronxCare Health System, Bronx, NY, 10457, USA;; 2 St. George’s University School of Medicine, University Centre Grenada, West Indies, Grenada;; 3 American University of the Caribbean School of Medicine, University Drive at Jordan Dr, Philipsburg, Cupecoy, Sint Maarten;; 4 Department of Internal Medicine, BronxCare Health System, Bronx, NY, 10457, USA

**Keywords:** Pericardial effusion, AL amyloidosis, ATTR amyloidosis, diagnosis, management, associations

## Abstract

**Background:**

Pericardial effusion is associated with amyloidosis, specifically amyloid light chain (AL) and transthyretin (ATTR) subtypes. However, the patients might present with different clinical symptoms.

**Objective:**

To determine the characteristics and associations of patients with pericardial effusion owing to either AL or ATTR amyloidosis.

**Methods:**

This study reviewed 26 studies from databases such as PubMed, MEDLINE, Web of Science, Google Scholar and CINAHL databases after protocol registration. The data were analyzed in IBM SPSS 21. Many statistical tests, such as Student *t*- and the Mann-Whitney U tests, were used. Multivariate logistic regression analysis was also performed. A *p*-value< 0.05 was considered significant.

**Results:**

A total of 531 patients with pericardial effusion secondary to amyloidosis were included. The mean age was 58.4±24.5 years. Most of the patients were male (72.9%). Common co-morbid conditions included hypertension (16.8%) and active smoking (12.9%). The most common time from symptom onset to the clinical presentation was less than 1 week (45%). ATTR amyloidosis was more common in older patients (*p*<0.05). Abdominal and chest discomfort were commonly associated with AL and ATTR amyloidosis, respectively (*p*<0.05). Patients with AL amyloidosis had a higher association with interventricular septal thickening and increased posterior wall thickness (*p*<0.05). First-degree atrioventricular block, left bundle branch block (LBBB), and atrial fibrillation (AF) were more associated with ATTR amyloidosis (*p*<0.05).

**Conclusion:**

Pericardial effusion in patients with AL amyloidosis was associated with hypertrophic remodeling, while conduction abnormalities were associated with ATTR amyloidosis.

## 1 INTRODUCTION

Amyloidosis is defined as amyloid deposition in various tissues and organs. Excess deposition of the proteins can lead to many complications. The amyloid light chain (AL) subtype is derived from excess immunoglobulin light chains, whereas the amyloid transthyretin (ATTR) subtype is a product of transthyretin, which is a precursor protein to albumin [1]. The global prevalence of amyloidosis is not well documented because the rarity of the condition leads to missed or delayed diagnoses. The estimated global 20-year prevalence of AL amyloidosis is 51 per million cases, while the wild-type ATTR amyloid was found to have a global prevalence of 172 per million cases [2, 3]. The United States prevalence of AL amyloidosis has been increasing. In 2007, the prevalence was found to be 15.5 cases per million, while in 2015 the prevalence was found to be 40.5 cases per million [4]. The prevalence in the United States of wild-type ATTR was found to be greater than 100,000 persons [5].

Both AL and ATTR amyloidosis can result in infiltrative heart disease known as cardiac amyloidosis (CA) [6]. Nearly all cases of CA can be attributed to these subtypes. Complications of CA include conduction problems and pericardial effusion [7]. Approximately one-half of the patients with CA develop pericardial effusion at some point in their lives [8]. The prevalence of AL-CA globally was found to be around 8 to 17 per 100,000 person-years, while ATTR-CA is unknown yet theorized to be underestimated [9]. Additionally, there is very little data regarding the prevalence of pericardial effusion in the United States. Pericardial effusion may also be a sign of myocardial edema following local amyloid infiltration and consecutive inflammation in patients with AL and ATTR [10]. The clinical presentation for pericardial effusion may vary from dyspnea and chest discomfort to hemodynamic instability. The management of pericardial effusion is dependent on size and presentation [11]. However, due to the systemic effects of amyloidosis, atypical symptoms might also be present.

As amyloidosis is a global systemic condition, the aim of the review is to determine clinical and transthoracic echocardiographic (TTE) characteristics in patients with pericardial effusion secondary to AL or ATTR amyloidosis. Additionally, the review aimed to determine associations of 12-lead electrocardiographic (ECG) and TTE parameters in both cohorts.

## 2 MATERIALS AND METHODS

### 2.1 Protocol Development and Systematic Review Registration

The protocol for the current systemic review was designed after a thorough literature review. The protocol was registered in PROSPERO (PROSPERO ID: CRD42023433913).

### 2.2 Search Strategy

The keywords (including all commonly used abbreviations of these terms) used in the search strategy were as follows: (“amyloidosis”[MeSH Terms] OR “amyloidosis”[All Fields] OR “amyloidoses”[All Fields]) AND (“pericardial effusion”[MeSH Terms] OR (“pericardial”[All Fields] AND “effusion” [All Fields]) OR “pericardial effusion”[All Fields]).

### 2.3 Data Extraction (Selection and Coding)

We screened PubMed, MEDLINE, Web of Science, Google Scholar and CINAHL databases for all case reports and observational studies of pericardial effusion associated with amyloidosis cases published from 1980 to 2023. The inclusion criteria were patients with pericardial effusions secondary to AL or ATTR amyloidosis, patients with a history of amyloidosis who developed effusions, and patients without any signs of renal dysfunction. The diagnosis of AL or ATTR amyloidosis was based on lab investigations and biopsy findings. The exclusion criteria included patients with renal dysfunction, patients with other etiologies responsible for the pericardial effusion or patients with AL or ATTR amyloidosis who did not develop any effusion. After the initial search, the duplicates were removed and imported, which included search studies to Rayyan software. Two independent reviewers screened the remaining studies for inclusion based on inclusion criteria, and researchers were blinded to each other's decisions. The screening was done *via* reading the abstract and full-text articles. Studies published in the English language or with an available english translation, were included in the initial review.

**Fig. (1):**
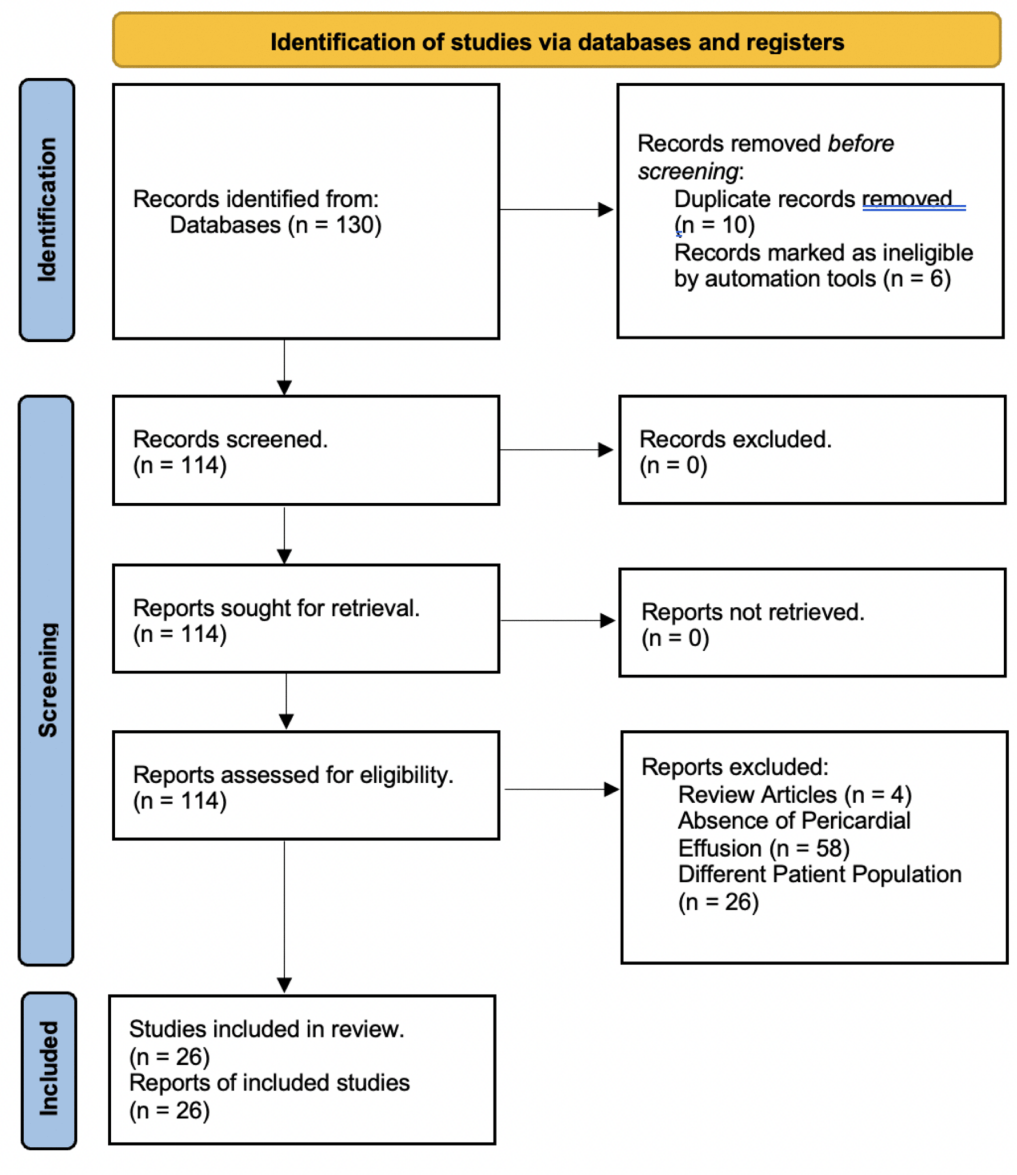
PRISMA diagram.

Using the PICO strategy, the patients who had been diagnosed with pericardial effusions secondary to AL or ATTR amyloidosis were included. There were no interventions studied. The outcome variables included age, gender, clinical features, electrocardiographic features, features on echocardiogram and mortality between the two cohorts. Data about study design, participant demographics, clinical presentation, symptoms, laboratory data, TTE data, clinical outcomes, and mortality were collected. One reviewer performed data extraction, and another reviewer cross-checked the extracted data for accuracy and completeness. The third reviewer resolved any disagreements. The study was excluded from the analysis if data could not be obtained. Publications that were not peer-reviewed were excluded from this study. Preferred Reporting Items for Systematic Reviews and Meta-Analyses (PRISMA) criteria were applied (Fig. **1**). The preliminary data were entered and recorded in an Excel spreadsheet.

### 2.4 Risk of Bias (Quality) Assessment

Quality assessment of all the included studies was assessed using the methodological quality and synthesis of case series and case reports described by Murad *et al*. (2018) (Tables **1** and **2**) [12]. Furthermore, observational studies were assessed using tools from the National Institute of Health [13].

Table **3** details the studies and their quality assessment.

### 2.5 Statistical Analysis

Statistical analysis was performed using IBM SPSS Statistics version 21 (IBM, Armonk, NY, USA). Continuous variables were expressed as mean ± standard deviation or median and interquartile range (IQR). Depending on the data distribution, Student *t*-, Mann-Whitney U or Kruskal-Wallis tests were applied. A chi-square test for categorical variables was performed. A *p*<0.05 was considered statistically significant. Logistic regression analysis was performed in both subgroups of amyloidosis. Variables with *p*< 0.2 were included in the final multivariate logistic regression analysis.

## 3 RESULTS

### 3.1 Overall Analysis of Patients with Amyloidosis and Pericardial Effusion

A total of 26 studies were analyzed. The details of the studies are described in Supplementary Tables **S1** and **S2** [6, 14-38]. There were a total of 531 patients whose data were included in the study. The clinical characteristics of the 531 patients are shown in Table **4**. The patients' mean age was 58.4±24.6 years, and most of the patients were male (387/531, 72.9%). Common co-morbid conditions include hypertension (16.8%), active smoking (12.9%), dyslipidemia (10.9%), AF (7.9%) and diabetes mellitus type II (DMT2) (6.5%). The most common time from symptom onset to the clinical presentation was less than 1 week (238/531, 45%) (Table **4**). All the cases of amyloidosis had been diagnosed on biopsy with amyloid deposits positive with Congo red staining or immunohistochemical stains positive for either disease.

Most of the patients had abdominal pain (59.5%). Recurrent effusions were seen in 6/531 cases (1.2%). Low voltage complexes were observed in 286/531 cases. Common TTE features included interventricular septal thickening (49.5%) and increased posterior wall thickness (48.9%). Fluid analysis was performed in 18/531 cases. Blood-stained effusions were observed in 17/531 cases. Medical management was preferred in most cases (98.20%). Out of 531 cases with pericardial effusion, 220 patients had died.

### 3.2 Comparison of Patients with Pericardial Effusion Secondary to AL amyloidosis and ATTR Amyloidosis

Table **5** shows the comparison of both subgroups of patients. Table **6** shows regression analysis for various variables for both cohorts.

AL amyloidosis was associated with male patients (*p*<0.05). Hypertension, smoking, DMT2 and dyslipidemia were associated with AL amyloidosis (*p*<0.05). Abdominal discomfort was commonly associated with AL amyloidosis (*p*<0.05). TTE features associated with AL amyloidosis included interventricular septal thickening and increased posterior wall thickness (*p*<0.05). Mortality was observed in 171/321 patients with pericardial effusion secondary to AL amyloidosis (*p*<0.05) however a uniform time frame was not present.

ATTR amyloidosis was more common in older patients and male gender (*p*<0.05). Peripheral neuropathy and coronary artery disease were associated with ATTR amyloidosis (*p*<0.05). Chest discomfort was associated with ATTR amyloidosis (*p*<0.05). First-degree atrioventricular block, left bundle branch block and AF were associated with ATTR amyloidosis (*p*<0.05). Right ventricular collapse on TTE was associated with ATTR amyloidosis (*p*<0.05). Mortality was observed in 49/210 patients with pericardial effusion secondary to ATTR amyloidosis (*p*>0.05); however, a uniform time frame was not present.

Survival analysis for patients with pericardial effusion secondary to AL or ATTR amyloidosis did not reveal any predictors in terms of clinical, electrocardiographic, or echocardiographic features for mortality.

## 4 DISCUSSION

In our systematic review, 531 cases were included based on the presence of either AL or ATTR amyloidosis, with findings diagnostic of pericardial effusion. The mean age of the patients was 58.4 ± 24.5 years, and the majority of the patients were male (387/531). Hypertension, smoking, DMT2 and dyslipidemia were significantly associated with AL amyloidosis and pericardial effusion. The time to presentation was variable, with common durations being acute (<1 week) or chronic (>6 months). Abdominal pain was significantly higher in patients with AL amyloidosis, whereas chest pain was significantly predominant in ATTR amyloidosis. Various arrhythmias were more commonly present in patients with ATTR amyloidosis. Additionally, 11/531 cases had cardiac tamponade [20, 26, 38].

The age is comparatively similar to another study with patients' age varying from 39 to 75 years [39]. Amyloidosis, specifically AL amyloidosis, is associated with a male predominance, as discussed in many studies [40-42]. The possible hypotheses for the male predominance include the cardioprotective impact of female sex hormones and larger cardiac anatomy in males [43, 44].

Interestingly, studies discussing the prevalence of comorbidities in AL amyloidosis mention renal disease, CHF and liver disease as the more commonly associated comorbidities compared to our results [45]. AL amyloidosis has a complicated pattern of cardiac and non-cardiac involvement. Prefibrillar oligomers and the direct cytotoxic effect of monoclonal chains can result in organ damage [46, 47]. Organs with pre-existing damage owing to pathogenic characteristics of comorbidities are at higher risk [46]. In fact, few studies discussed patients with AL amyloidosis having a reduction of NT-proBNP, a marker of cardiac dysfunction, after chemotherapy. However, the cardiac deposits remained unaltered, and patients had improved cardiac function, with a decreased likelihood of pericardial effusion [48, 49].

The bimodal distribution of time to clinical presentation was possibly linked to the progression of pericardial effusion in patients with amyloidosis in the absence of interventions and presenting as acute emergencies [19, 26, 37]. Additionally, in non-emergent cases, the time was relatively similar to another study [16, 17, 50].

In a study discussing patients with AL amyloidosis, abdominal pain was observed in 50% of the patients and resulted in biopsy-proven systemic diagnosis in many patients [51]. Additionally, cardiac involvement was also observed in the same cohort of patients [52]. Patients with pericardial effusion and AL amyloidosis had significantly greater interventricular septal thickening and posterior wall thickness. Although similar results have been reported previously, patients with ATTR amyloidosis were noted to have significantly greater measurements of the same variables [51]. AL amyloidosis has a greater ratio of deposition of amyloid plaques, resulting in hypertrophy and diastolic dysfunction [53]. Pericardial effusion in AL amyloidosis is associated with hypertrophic abnormalities, as observed in our findings. While these abnormalities might predispose to heart failure, these findings might not lead to poor outcomes or prognosis in patients with AL amyloidosis as opposed to the presence of pleural effusions discussed by studies as a marker for possible poor prognosis [6].

ATTR amyloidosis was more common in older patients, with a previous study reporting a mean age > 70 years [54]. Previous studies have discussed AF as commonly being present in patients with ATTR amyloidosis [55, 56]. Right ventricular collapse on TTE was significantly associated with ATTR amyloidosis in patients with pericardial effusion. It has been postulated that TTR molecules despite having a similar pathogenic potential, can additionally also predispose patients to fluid overload that can present as effusion [57]. Cardiac involvement in patients with ATTR amyloidosis can result in mortality because of increased hospitalizations because of heart failure [58]. However, conduction abnormalities can further complicate these hospitalizations and result in poor outcomes.

The pathogenesis of pericardial effusion as a complication of AL or ATTR amyloidosis has been hypothesized in literature. The deposition of AL or ATTR-based chains on the pericardial surface might result in a fluid buildup similar to that observed in pleural effusion that has been studied in a few cases [59]. Left ventricular dilatation that might occur secondary to the deposition of these plaques can cause persistent localized inflammation, leading to pericardial effusion [14]. Plasma cells migrating to these sites might also lead to chronic inflammation, resulting in pericardial effusion [14].

Our review's major strengths are the inclusion of all relevant cases to date and an in-depth comparison of clinical, TTE and diagnostic features of pericardial effusion in patients with AL or ATTR amyloidosis. The limitations include the retrospective nature of the study, the absence of laboratory data in studies and a lack of data about other amyloidosis categories. Additionally, the study aimed to explore pericardial effusion as a complication of AL or ATTR amyloidosis, as compared to establishing prevalence. Furthermore, the size of the effusion was a qualitative variable and might not have necessarily been on a uniform scale due to the heterogeneity of the included cases.

## CONCLUSION

Pericardial effusion is one of the complications of patients with AL and ATTR amyloidosis. The pathogenesis of the pericardial effusion in both cohorts has been hypothesized, with pericardial effusion in patients with AL amyloidosis being associated with hypertrophic remodeling. Conduction abnormalities were associated with ATTR amyloidosis. Abdominal discomfort was frequently linked with AL amyloidosis. TTE characteristics indicative of AL amyloidosis comprised thickening of the interventricular septum and an increase in posterior wall thickness. First-degree atrioventricular block, left bundle branch block, and atrial fibrillation were identified as associations with ATTR amyloidosis. Additionally, right ventricular collapse observed on TTE was found to be correlated with ATTR amyloidosis. Understanding the characteristics of these cohorts could provide further evidence-based interventions in patients with amyloidosis. Further studies are warranted to confirm the findings of our review.

## Figures and Tables

**Table 1:** Tool for evaluating the methodological quality of case reports and case series.

**Domains**	**Leading Explanatory Questions**	**Question used**
Selection	1. Does the patient(s) represent(s) the whole experience of the investigator (centre) or is the selection method unclear to the extent that other patients with similar presentation may not have been reported?	Yes
Ascertainment	2. Was the exposure adequately ascertained? 3. Was the outcome adequately ascertained?	Yes Yes
Causality	4. Were other alternative causes that may explain the observation ruled out? 5. Was there a challenge/rechallenge phenomenon? 6. Was there a dose–response effect? 7. Was follow-up long enough for outcomes to occur?	Yes No No Yes
Reporting	8. Is the case(s) described with sufficient details to allow other investigators to replicate the research or to allow practitioners make inferences related to their own practice?	Yes

**Table 2:** Quality assessment tool for observational cohort and cross-sectional studies.

**Criteria**	**Question used**
1. Was the research question or objective in this paper clearly stated?	Yes
2. Was the study population clearly specified and defined?	Yes
3. Was the participation rate of eligible persons at least 50%?	Yes
4. Were all the subjects selected or recruited from the same or similar populations (including the same time period)? Were inclusion and exclusion criteria for being in the study prespecified and applied uniformly to all participants?	Yes
5. Was a sample size justification, power description, or variance and effect estimates provided?	Yes
6. For the analyses in this paper, were the exposure(s) of interest measured prior to the outcome(s) being measured?	No
7. Was the timeframe sufficient so that one could reasonably expect to see an association between exposure and outcome if it existed?	Yes
8. For exposures that can vary in amount or level, did the study examine different levels of the exposure as related to the outcome (*e.g.*, categories of exposure, or exposure measured as continuous variable)?	No
9. Were the exposure measures (independent variables) clearly defined, valid, reliable, and implemented consistently across all study participants?	Yes
10. Was the exposure(s) assessed more than once over time?	No
11. Were the outcome measures (dependent variables) clearly defined, valid, reliable, and implemented consistently across all study participants?	Yes
12. Were the outcome assessors blinded to the exposure status of participants?	Yes
13. Was loss to follow-up after baseline 20% or less?	Yes
14. Were key potential confounding variables measured and adjusted statistically for their impact on the relationship between exposure(s) and outcome(s)?	Yes

**Table 3:** Quality assessment of included studies.

**Judgment**	**%**	**Number**	**Case reports**	**Case series**	**Observational Studies**
Good	92.3	24	17	3	4
Fair	7.7	2	0	1	1
Poor	0	0	0	0	0

**Table 4:** Baseline characteristics of 531 patients.

**Variables**	**n (%)**
Age in years (Mean±S.D)	58.4±24.6
Asymptomatic	198 (37.4)
Gender	-
Male	387 (72.9)
Female	144 (27.1)
Time till Presenting Symptoms	-
Less than 1 week	228 (45.0)
>1 week and <1 month	1 (0.2)
>1 month and <3 months	1 (0.2)
>3 months	2 (0.4)
>6 months	90 (16.8)
Comorbid Conditions	-
HTN	90 (16.8)
Smoking	68 (12.9)
DLD	58 (10.9)
History of AF	42 (7.9)
DMT2	35 (6.5)
Peripheral Neuropathy	27 (5.2)
CAD	19 (3.6)
CVD	2 (0.4)
Ethanol Abuse	1 (0.2)
CHF	1 (0.2)
Epilepsy	1 (0.2)
Nephrotic Syndrome	1 (0.2)
Cardiomyopathy	1 (0.2)
Symptoms at Presentation	-
Abdominal Pain	198 (59.5)
Dyspnea on Exertion	61 (18.5)
Chest Pain	54 (16.1)
Leg Swelling	9 (2.8)
Fatigue	3 (1.0)
Arthralgia	3 (1.0)
Pallor	1 (0.3)
Syncope	1 (0.3)
Weight Loss	1 (0.3)
Periorbital Swelling	1 (0.3)
AL amyloidosis	321 (60.4)
ATTR amyloidosis	210 (39.6)
Electrocardiographic Features	-
Low Voltage Complexes	301 (56.7)
Ischemic Changes	91 (17.2)
First-degree AV block	54 (10.3)
LBBB	52 (9.9)
AF	45 (8.5)
Echocardiographic Features	-
Dilated Left Ventricle	167 (31.5)
Interventricular Septum Thickening	263 (49.5)
Global Hypokinesis	1 (0.2)
Myocardial Speckling	111 (21.0)
Left Ventricular Hypertrophy	240 (45.2)
Right Ventricular Collapse	1 (0.2)
Increased Posterior Wall Thickness	260 (49.0)
Preserved Systolic Function	67 (12.7)
Atrial Dilatation	4 (0.8)
Increased Atrial Septal Thickness	11 (2.0)
Right Atrial Collapse	2 (0.4)
Mitral Regurgitation	1 (0.2)
Pulmonary Hypertension	1 (0.2)
Dilated Inferior Vena Cava	1 (0.2)

**Abbreviations:** S.D-Standard Deviation, HTN-hypertension, DLD-dyslipidemia, AF-atrial fibrillation, DMT2-type 2 diabetes mellitus, CAD-coronary artery disease, CVD-cerebrovascular disease, CHF-congestive heart failure, AL-light chain, ATTR-transthyretin, AV-atrioventricular, LBBB-left bundle branch block, AF-atrial fibrillation

**Table 5:** Characteristics of patients with pericardial effusions due to AL amyloidosis and ATTR amyloidosis.

**Variables**	**AL Amyloidosis [n (%)], Total N-321**	**ATTR Amyloidosis [n (%)], Total N-210**	***p-*value**
Age in years (Mean±SD)	57.8±15.3	81.5±12.0	0.00
Gender	-	-	0.00
Male	284 (88.5)	103 (49.0)	
Female	37 (11.5)	107 (51.0)	
Asymptomatic	112 (34.9)	86 (40.9)	0.08
Time till Presenting Symptoms	95 (29.6)	119 (56.7)	0.97
Less than 1 week	188 (58.6)	40 (19.1)	
>1 week and <1 month	1 (0.3)	0 (0.0)	
>1 month and <3 months	1 (0.3)	0 (0.0)	
>3 months	1 (0.3)	1 (0.5)	
>6 months	2 (0.6)	88 (0.4)	
Comorbid Conditions	-	-	-
HTN	51 (15.9)	34 (16.2)	0.03
Smoking	38 (11.8)	27 (12.9)	0.00
DLD	32 (9.9)	23 (10.9)	0.00
History of AF	15 (4.7)	25 (11.9)	<0.05
T2DM	18 (5.6)	15 (7.1)	0.00
Peripheral Neuropathy	11 (3.4)	15 (7.1)	0.00
CAD	7 (2.2)	11 (5.2)	0.00
CVD	2 (0.6)	0 (0.0)	0.98
Ethanol Abuse	0 (0.0)	1 (0.5)	0.89
CHF	0 (0.0)	1 (0.5)	0.98
Epilepsy	1 (0.3)	0 (0.0)	0.97
Nephrotic Syndrome	1 (0.3)	0 (0.0)	0.97
Cardiomyopathy	1 (0.3)	0 (0.0)	0.97
Symptoms at Presentation	-	--	
Abdominal Pain	118 (36.8)	70 (33.3)	0.02
Dyspnea on Exertion	1 (0.3)	57 (27.1)	0.25
Chest Pain	0 (0.0)	51 (24.5)	0.03
Leg Swelling	2 (0.6)	7 (3.3)	0.98
Fatigue	2 (0.6)	1 (0.5)	0.14
Arthralgia	2 (0.6)	1 (0.5)	0.14
Pallor	1 (0.3)	0 (0.0)	0.97
Syncope	1 (0.3)	0 (0.0)	0.97
Weight Loss	0 (0.0)	1 (0.5)	0.97
Periorbital Swelling	1 (0.3)	0 (0.0)	0.97
Electrocardiographic Features	-	-	-
Low Voltage Complexes	240 (74.8)	46 (21.9)	0.83
Ischemic Changes	2 (0.6)	85 (40.5)	0.27
First-degree AV block	2 (0.6)	50 (23.8)	0.03
LBBB	1 (0.3)	48 (22.9)	<0.05
AF	0 (0.0)	43 (20.5)	0.03
Echocardiographic Features	-	-	-
Dilated Left Ventricle	74 (23.1)	85 (40.5)	0.19
Interventricular Septum Hypertrophy	162 (50.5)	88 (41.9)	0.04
Global Hypokinesis	1 (0.3)	0 (0.0)	0.97
Myocardial Speckling	14 (4.4)	92 (43.8)	0.76
Left Ventricular Hypertrophy	228 (95.0)	12 (5.0)	0.49
Right Ventricular Collapse	0 (0.0)	1 (0.5)	0.03
Increased Posterior Wall Thickness	125 (38.9)	122 (58.1)	0.03
Preserved Systolic Function	63 (19.6)	1 (0.5)	0.35
Atrial Dilatation	3 (0.9)	1 (0.5)	0.95
Increased Atrial Septal Thickness	10 (3.1)	0 (0.0)	0.87
Right Atrial Collapse	2 (0.6)	0 (0.0)	0.87
Mitral Regurgitation	1 (0.3)	0 (0.0)	0.97
Pulmonary Hypertension	1 (0.3)	0 (0.0)	0.97
Dilated Inferior Vena Cava	1 (0.3)	0 (0.0)	0.97

**Abbreviations:** S.D-Standard Deviation, HTN-hypertension, DLD-dyslipidemia, AF-atrial fibrillation, DMT2-type 2 diabetes mellitus, CAD-coronary artery disease, CVD-cerebrovascular disease, CHF-congestive heart failure, AL-light chain, ATTR-transthyretin, AV-atrioventricular, LBBB-left bundle branch block, AF-atrial fibrillation

**Table 6:** Regression analysis for electrocardiographic and echocardiographic parameters observed in patients with pericardial effusion in AL and ATTR Amyloidosis.

**Variables**	**AL Amyloidosis**			**ATTR Amyloidosis**		
	**Odds Ratio**	**95% CI**	***p*-value**	**Odds Ratio**	**95% CI**	***p*-value**
First-degree AV block	0.3	0.0-4.1	<0.05	2.4	0.2-3.8	0.04
Left Bundle Branch Block	NA	NA	NA	1.1	0.0-2.3	0.01
AF	NA	NA	NA	1.3	0.0-7.6	0.03
IVS Thickening	1.1	0.0-2.9	<0.05	0.3	0.0-1.8	>0.05
Increased Posterior Wall Thickness	1.1	0.0-3.5	0.011	0.5	0.1-2.6	0.015

## Data Availability

The data that support the findings of this study are available from the corresponding author, [N.J.], upon reasonable request.

## LIST OF ABBREVIATIONS

CA: Cardiac Amyloidosis
IQR: Interquartile Range
LBBB: Left Bundle Branch Block
TTE: Transthoracic Echocardiographic

## STANDARDS OF REPORTING

PRISMA guidelines and methodology were followed.

## SUPPLEMENTARY MATERIAL

PRISMA checklist is available as supplementary material on the publisher’s website along with the published article.
