# Supplementary material for: Associations of Patients with Pericardial Effusion Secondary to Light-Chain or Transthyretin Amyloidosis- A Systematic Review
Source: Curr Cardiol Rev. 2024 Mar 8;20(4):E080324227805. doi: 10.2174/011573403X280737240221060630 (PMC11327831; doi:10.2174/011573403X280737240221060630)
Supplement: Supplementary file 1 [file CCR-20-E080324227805_SD1.pdf]

Supplementary Material

Associations of Patients with Pericardial Effusion Secondary to Light-Chain or Transthyretin Amyloidosis- A Systematic Review

Nismat Javed[1,*], Kirit Singh[2], Justin Shirah[3] and Timothy J. Vittorio[4]

[1]Department of Internal Medicine, BronxCare Health System, Bronx, NY, 10457, USA; [2]St. George's University School of Medicine, University Centre Grenada, West Indies, Grenada; [3]American University of the Caribbean School of Medicine, 1 University Drive at Jordan Dr, Philipsburg, Sint Maarten; [4]Department of Cardiology, BronxCare Health System, Bronx, NY, 10457, USA

Section and Topic	Item #	Checklist item	Location where item is reported
**TITLE**			
Title	1	Identify the report as a systematic review.	Title
**ABSTRACT**			
Abstract	2	See the PRISMA 2020 for Abstracts checklist.	As per guidelines
**INTRODUCTION**			
Rationale	3	Describe the rationale for the review in the context of existing knowledge.	1st 2 paragraphs of intro
Objectives	4	Provide an explicit statement of the objective(s) or question(s) the review addresses.	Last paragraph of intro
**METHODS**			
Eligibility criteria	5	Specify the inclusion and exclusion criteria for the review and how studies were grouped for the syntheses.	1st paragraph under data extraction
Information sources	6	Specify all databases, registers, websites, organisations, reference lists and other sources searched or consulted to identify studies. Specify the date when each source was last searched or consulted.	1st paragraph under data extraction
Search strategy	7	Present the full search strategies for all databases, registers and websites, including any filters and limits used.	Under search strategy
Selection process	8	Specify the methods used to decide whether a study met the inclusion criteria of the review, including how many reviewers screened each record and each report retrieved, whether they worked independently, and if applicable, details of automation tools used in the process.	1st paragraph under data extraction
Data collection process	9	Specify the methods used to collect data from reports, including how many reviewers collected data from each report, whether they worked independently, any processes for obtaining or confirming data from study investigators, and if applicable, details of automation tools used in the process.	All of information under data extraction
Data items	10a	List and define all outcomes for which data were sought. Specify whether all results that were compatible with each outcome domain in each study were sought (e.g. for all measures, time points, analyses), and if not, the methods used to decide which results to collect.	All of information under data extraction
	10b	List and define all other variables for which data were sought (e.g. participant and intervention characteristics, funding sources). Describe any assumptions made about any missing or unclear information.	All of information under data extraction
Study risk of bias assessment	11	Specify the methods used to assess risk of bias in the included studies, including details of the tool(s) used, how many reviewers assessed each study and whether they worked independently, and if applicable, details of automation tools used in the process.	Under bias assessment
Effect measures	12	Specify for each outcome the effect measure(s) (e.g. risk ratio, mean difference) used in the synthesis or presentation of results.	N/A, not a meta-analysis
Synthesis methods	13a	Describe the processes used to decide which studies were eligible for each synthesis (e.g. tabulating the study intervention characteristics and comparing against the planned groups for each synthesis (item #5)).	All of information under data extraction

Section and Topic	Item #	Checklist item	Location where item is reported
	13b	Describe any methods required to prepare the data for presentation or synthesis, such as handling of missing summary statistics, or data conversions.	Under statistical analysis
	13c	Describe any methods used to tabulate or visually display results of individual studies and syntheses.	Under statistical analysis
	13d	Describe any methods used to synthesize results and provide a rationale for the choice(s). If meta-analysis was performed, describe the model(s), method(s) to identify the presence and extent of statistical heterogeneity, and software package(s) used.	Under statistical analysis
	13e	Describe any methods used to explore possible causes of heterogeneity among study results (e.g. subgroup analysis, meta-regression).	N/A, not a meta-analysis
	13f	Describe any sensitivity analyses conducted to assess robustness of the synthesized results.	N/A, not a meta-analysis
Reporting bias assessment	14	Describe any methods used to assess risk of bias due to missing results in a synthesis (arising from reporting biases).	Under bias assessment
Certainty assessment	15	Describe any methods used to assess certainty (or confidence) in the body of evidence for an outcome.	N/A
**RESULTS**			
Study selection	16a	Describe the results of the search and selection process, from the number of records identified in the search to the number of studies included in the review, ideally using a flow diagram.	Figure 1
	16b	Cite studies that might appear to meet the inclusion criteria, but which were excluded, and explain why they were excluded.	Figure 1
Study characteristics	17	Cite each included study and present its characteristics.	Supplementary Tables 1 and 2
Risk of bias in studies	18	Present assessments of risk of bias for each included study.	Table 3
Results of individual studies	19	For all outcomes, present, for each study: (a) summary statistics for each group (where appropriate) and (b) an effect estimate and its precision (e.g. confidence/credible interval), ideally using structured tables or plots.	Table 4
Results of syntheses	20a	For each synthesis, briefly summarise the characteristics and risk of bias among contributing studies.	N/A, not a meta-analysis
	20b	Present results of all statistical syntheses conducted. If meta-analysis was done, present for each the summary estimate and its precision (e.g. confidence/credible interval) and measures of statistical heterogeneity. If comparing groups, describe the direction of the effect.	N/A, not a meta-analysis
	20c	Present results of all investigations of possible causes of heterogeneity among study results.	N/A, not a meta-analysis
	20d	Present results of all sensitivity analyses conducted to assess the robustness of the synthesized results.	N/A, not a meta-analysis
Reporting biases	21	Present assessments of risk of bias due to missing results (arising from reporting biases) for each synthesis assessed.	N/A, not a meta-analysis
Certainty of evidence	22	Present assessments of certainty (or confidence) in the body of evidence for each outcome assessed.	N/A, not a meta-analysis
**DISCUSSION**			
Discussion	23a	Provide a general interpretation of the results in the context of other evidence.	Under discussion
	23b	Discuss any limitations of the evidence included in the review.	Last paragraph of discussion
	23c	Discuss any limitations of the review processes used.	Last paragraph of discussion
	23d	Discuss implications of the results for practice, policy, and future research.	Under conclusions
**OTHER INFORMATION**			
Registration and protocol	24a	Provide registration information for the review, including register name and registration number, or state that the review was not registered.	Under protocol development

Section and Topic	Item #	Checklist item	Location where item is reported
	24b	Indicate where the review protocol can be accessed, or state that a protocol was not prepared.	Under protocol development
	24c	Describe and explain any amendments to information provided at registration or in the protocol.	N/A
Support	25	Describe sources of financial or non-financial support for the review, and the role of the funders or sponsors in the review.	N/A
Competing interests	26	Declare any competing interests of review authors.	None
Availability of data, code and other materials	27	Report which of the following are publicly available and where they can be found: template data collection forms; data extracted from included studies; data used for all analyses; analytic code; any other materials used in the review.	N/A

Supplementary Table 1. Summary of cases reviewed and their demographic characteristics.

Author	Number of Patients	Age	Gender	Comorbidities	Diagnosis	Presenting Symptoms
Binder [6]	143	74	56 males, 87 females	Polyneuropathy (33.3), Diabetes mellitus (10.3), atrial fibrillation (48.7), coronary artery disease (23.1), intracardiac device (14.1)	85 patients with ATTR and 58 patients with AL.	None
Itagaki [14]	1	40	Male	Epilepsy and cerebral palsy	AL amyloidosis	Pallor
Yuda [15]	14	59	7 males, 7 females	NA	ATTR amyloidosis	NA
John [16]	1	51	Female	None	AL amyloidosis	Chest pain, dyspnea, syncope
Matute-Blanco [17]	1	90	Male	dyslipidemia, atrial fibrillation and ischemic stroke	ATTR amyloidosis	Dyspnea
Damy [18]	198	68	131 males, 67 females	Hypertension, Dyslipidemia, Diabetes, Smoking	AL (n 118) m-TTR (n 57) WT-TTR (N 23)	NA
Toyama [19]	1	73	Female	Myomectomy	AL and ATTR both	Dyspnea
Wang [20]	9	56	8 males, 3 females	NA	AL	NA
Scafi [21]	1	35	Male	NA	AL	Abdominal pain
Georgin-Lavialle [22]	1	61	Male	Hypertension, hyperlipidemia	AL	Fatigue, anthralgia, weight loss
Barros-Gomes [23]	1	49	Male	NA	AL	Periorbital rash
Abdelazeem [24]	1	72	Female	NA	AL	Dyspnea,orthopnea, leg swelling
Baca [25]	1	83	Female	Diabetes and hypertension	AL	Dyspnea and leg swelling
Kuprian [26]	1	73	Female	Nephrotic syndrome	AL	Dyspnea
Hirata [27]	1	40	Male	NA	AL	Dyspnea
Yamamoto [28]	1	60	Male	Embolic stroke, hypertrophic cardiomyopathy	ATTR	Carpal tunnel syndrome
Medvedovsky	43	62	42 males	Atrial fibrillation and pleural	AL	NA

[29]				effusion		
Hsu [30]	7	61	6 males, 1 female	NA	ATTR amyloidosis	peripheral neuropathy, resultant walking difficulty (5/7, 71%) and/or functional impairment of the hands (3/7, 43%)
Mekinian [31]	6	63	19 males, 10 females	NA	AL amyloidosis	NA
Domingues [32]	1	43	male	NA	AL amyloidosis	Fatigue, bleeding gums and anthralgia
Huang [33]	42	NA	21 males, 21 females	NA	Unspecified	chest tightness and shortness of breath (37 cases, 88.1%), chest pain (20 cases, 47.6%), right heart failure (27 cases, 64.3%), fatigue (27 cases, 64.3%), renal insufficiency and proteinuria (30 cases, 71.4%)
Cheng [34]	1	73	Female	NA	AL amyloidosis	Dyspnea
Sekiya [35]	1	48	male	ethanol abuse	Unspecified	upper abdominal and lower, chest pain.
Qian [36]	52	53	65 males	NA	AL amyloidosis	NA
Lavine [37]	1	67	female	diabetes, hypertension, atrial fibrillation, congestive heart failure, and a recent	Unspecified	shortness of breath, a syncopal episode, declining mental status
Navarro [38]	1	64	male	NA	Unspecified	fatigue, chest pain, dyspnea

Supplementary Table 2. Summary of clinical, electrocardiographic and echocardiographic characteristics.

Author	Time of Presentation	Electrocardiography	Imaging	Size	Management	Mortality
Binder [6]	None	Not specified	Not specified	Small	None	28 patients with AL and 14 patients ATTR died
Itagaki [14]	On arrival	Not specified	Dilated left ventricle	Large and recurrent	Pericardial resection and fenestration	Alive
Yuda [15]	Not specified	Not specified	Not specified	Not specified	Not specified	Alive
John [16]	2 months	Not specified	Global hypokinesia, mild mitral regurgitation, and myocardial ‘sparkling’	Large and chronic	Pericardiocentesis	Alive
Matute-Blanco [17]	6 months	Not specified	Severe left ventricular hypertrophy with preserved systolic function, severe biatrial dilatation, severe pulmonary hypertension, and severe asymmetric PD without right chamber collapse	Large and recurrent	Pericardiocentesis and percutaneous balloon pericardiotomy	Alive
Damy [18]	Not specified	Not specified	IVST and PWTd were	Small	Not specified	67 deaths

			abnormally high in all types of amyloidosis. LV hypertrophy was more marked in the TTR groups than in the AL group			
Toyama [19]	6 months	Not specified	Not specified	Large and recurrent	Pericardiocentesis	Dead
Wang [20]	Not specified	Low voltage complexes	increased interventricular septum thickness (>1.3 cm) and(or) left ventricular lateral wall thickness (>1.1 cm). Increased thickness of the atrial septum was also found (thickness at end diastole >0.6 cm) in 5 patients, and 7 patients had enlarged atrial internal diameter. Myocardial echoes of interventricular septum and free wall of left ventricle were enhanced, and 6 had "ground glass" or "sparkling" appearance of walls Mild-moderate hydropericardium was found in 9 patients, and pleural effusion in 10 patients. Five patients had depressed left ventricular systolic function (LVEF<50%), and 10 had a restrictive left ventricular filling pattern	Hemopericardium in 9 cases	Not specified	Alive
Scafi [21]	1 week	Low voltage complexes	Preserved ejection fraction	Recurrent and large	On dialysis and possible transplant	Alive
Georgin-Lavialle [22]	Not specified	Low voltage complexes	large circumferential pericardial effusion with right atrial compression	Large	Surgical evacuation and therapy rituximab, bendamustine, and dexamethasone for six cycles.	Alive
Barros-Gomes [23]	8 weeks	Not specified	granular 'sparkling' appearance of myocardium, biventricular wall thickening, and thickening of interatrial septum and valves; severe diastolic dysfunction with moderately reduced LV ejection fraction; mild decrease in right ventri-	Small	Not specified	Alive

			cle systolic function; and small pericardial effusion			
Abdelazeem [24]	Months	Prolonged PR interval, type one atrioventricular block	left ventricular (LV) ejection fraction of 25%-30%, severely increased left atrial volume 48.6 ml/m2, moderate pulmonary hypertension with right ventricular systolic pressure of 53.66 mmHg, and a moderate to large pericardial effusion behind LV without tamponade	Moderate	Pericardial window and bortezomib, cy-clophosphamide, and dexamethasone thera-py	Alive
Baca [25]	1 week	left axis deviation, T-wave inversions in V5–6, I and aVL, biphasic T waves in V3–4, left anterior fascicular block, low-voltage QRS complexes, normal intervals, no hypertrophy, and reduced R wave progression	severe concentric ven-tricular hypertrophy and interventricular septal wall thickening with ‘‘sparkling’’ hyperechoic appearance of the myocardium, mildly reduced left ventricular ejection fraction, left atrial dilatation, and small pericardial effusion. The left ventricular posterior wall diameter measured 22 mm and the interventricular septum diameter meas-ured 27 mm	Moderate	Medical therapy	Alive
Kuprian [26]	5 days	low voltage with poor R wave progression, with-out ST or T wave chang-es	moderate-sized effusion with compression of the right atrium and ven-tricular free wall	Large and recurrent	Pericardiocentesis and dexamethasone and bortezomib	Dead
Hirata [27]	2 months	low-amplitude R-wave on the limb leads and a QS pattern on leads V1–V3	increased left ventricu-lar (LV) wall thickness with a small pericardial effusion. Thickening of the right ventricular (RV) wall was also shown. Left ventricular systolic function was preserved, with an ejec-tion fraction (EF) of 63%. Longitudinal myocardial systolic strain based on two-dimensional speckle-tracking echocardiog-raphy showed far more significant LV dysfunc-tion. Global longitudi-nal strain was markedly	Small	Bortzemib and dexa-methasone, auto-PBSCT	Alive

			reduced to −6.2%, and bull's eye mapping revealed the characteristic apical sparing pattern. The regional values of the RV free wall longitudinal strain were reduced to −7.0%. Left atrial (LA) strain was also reduced to −14%. Doppler e' values were low, with an E/e' ratio >15. Pulmonary artery systolic pressure could not be estimated due to absence of tricuspid regurgitation. Inferior vena cava (IVC) was dilated and showed decreased respiratory variability.			
Yamamoto [28]	Not specified	normal sinus rhythm with QS waves in inferior leads, and with low QRS voltages in leads V1 to V4	Severe asymmetric left ventricular hypertrophy, biatrial dilatation, pericardial effusion, and preserved left ventricular ejection fraction of 50% with severe diastolic dysfunction.	Small	Diuretics and tafadamis	Alive
Medvedovsky [29]	180 days	36 had low QRS voltage, 24 had pseudoinfarct pattern, 5 had AV block and 10 had LBBB	LVESD 3.0, 27 had granular speckling pattern	Not specified	Not specified	16 deaths, rest alive
Hsu [30]	Not specified	AV block (5/7, 71%), and bundle-branch block (BBB; 5/7, 71%	pericardial effusion associated with progressive ventricular wall thickening, two patients had low voltage QRS in the limb leads and the precordial leads, respectively. Echocardiogram a granular sparkling appearance of the ventricular myocardium in four patients. Concentric LV hypertrophy was noted in 4 patients	Not specified	Not specified	Alive
Mekinian [31]	Not specified	4 had low voltage EKG,	Not specified	Not specified	Not specified	15 deaths
Domingues [32]	2 months	sinus tachycardia and low voltage complexes	biventricular hypertrophy with preserved ejection fraction, restrictive physiology with elevated filling pressures and relative "apical sparing" on 2D longitudinal strain, thickened interatrial septum and atrioven-	Small	Chemotherapy	Alive

			tricular valves and a small pericardial effusion			
Huang [33]	Chronic	low voltage in limb leads (32 cases, 76.2%), R wave Dystrophy (29 cases, 69.0%), ST-T changes (17 cases, 40.5%), pseudo-necrotic Q waves (28 cases, 66.7%) and various arrhythmias (36 cases, 85.7%)	42 cases (100%) all had different degrees of left ventricular posterior wall or interventricular septal thickening, cardiac granule-like flash echo enhancement or ground-glass-like changes, and left atrial hypertrophy; 36 cases (85.7%) had little to medium pericardial Effusion, 27 cases (64.3%) with decreased left ventricular ejection fraction	Moderate	Not specified	Alive
Cheng [34]	Chronic	Not specified	left ventricular hypertrophy and an ejection fraction of 43%. moderately impaired biventricular systolic function globally (left ventricular ejection fraction 43% – normal range 57–81%; right ventricular ejection fraction 45% – normal range 53–73%), thickened atrioventricular valves and mild mitral regurgitation. Left ventricular mass (165 g – normal range 42–150 g) and mass index (104 g/m2 – normal range 31–79 g/m2) were There was a restrictive filling pattern with elevated left-sided filling pressures, left atrial enlargement and mild mitral regurgitation. The right heart was normal with normal filling pressures. increased with marked asymmetrical hypertrophy, while right ventricular volumes and mass were normal. The atria were enlarged with increased atrial septal thickness	Small	Chemotherapy	Death

			(10 mm). Delayed enhancement imaging, a technique to detect areas of increased extracellular space, demonstrated widespread and predominantly subendocardial hyperenhancement in all myocardial segments of both ventricles, as well as the inter-atrial and interventricular septa – findings consistent with a generalised infiltrative process. The total hyperenhancing mass in the left ventricle was quantified at 42 g			
Sekiya [35]	7 months	left bundle branch block	Not specified	Small	Mephalan	Death
Qian [36]	Not specified	48 cases had low voltage complexes	Increased IVS	Not specified	Not specified	91 deaths
Lavine [37]	1 day	atrial fibrillation with a rapid ventricular response rate of 144 beats/min, low voltage QRS complexes and a non-specific intraventricular conduction delay	right and left ventricular hypertrophy with poor left ventricular systolic function, and a large pericardial effusion. The myocardium demonstrated a granular appearance. There was no right ventricular diastolic collapse	Large	Pericardiocentesis	Alive
Navarro [38]	Chronic	Not specified	Not specified	Not specified	Pericardiocentesis and pericardectomy	Alive
